# A Molecular Determinant of West Nile Virus Secretion and Morphology as a Target for Viral Attenuation

**DOI:** 10.1128/JVI.00086-20

**Published:** 2020-06-01

**Authors:** Justine Basset, Julien Burlaud-Gaillard, Maxence Feher, Philippe Roingeard, Félix A. Rey, Nathalie Pardigon

**Affiliations:** a Institut Pasteur, Arbovirus Group, Environment and Infectious Risks Unit, Paris, France; b Université Paris Diderot, Sorbonne Paris Cité, Cellule Pasteur, Paris, France; c Plateforme IBISA de Microscopie Electronique, PST ASB, Université de Tours and CHRU de Tours, Tours, France; d INSERM U1259, Université de Tours and CHRU de Tours, Tours, France; e Laboratory for Urgent Response to Biological Threats, Institut Pasteur, Paris, France; f Institut Pasteur, Structural Virology Unit, Paris, France; g CNRS UMR 3569, Virologie, Paris, France; University of North Carolina at Chapel Hill

**Keywords:** West Nile virus, flavivirus, attenuation, secretion, membrane protein

## Abstract

West Nile virus (WNV), a member of the *Flavivirus* genus and currently one of the most common arboviruses worldwide, is associated with severe neurological disease in humans. Its high potential to reemerge and rapidly disseminate makes it a bona fide global public health problem. The surface membrane glycoprotein (M) has been associated with *Flavivirus*-induced pathogenesis. Here, we identified a key amino acid residue at position 36 of the M protein whose mutation impacts WNV secretion and promotes viral attenuation. We also identified a compensatory site at position M-43 whose mutation stabilizes M-36 substitution both *in vitro* and *in vivo*. Moreover, we found that introduction of the two mutations together confers a full attenuation phenotype and protection against wild-type WNV lethal challenge, eliciting potent neutralizing-antibody production in mice. Our study thus establishes the M protein as a new viral target for rational design of attenuated WNV strains.

**IMPORTANCE** West Nile virus (WNV) is a worldwide (re)emerging mosquito-transmitted *Flavivirus* causing fatal neurological diseases in humans. However, no human vaccine has been yet approved. One of the most effective live-attenuated vaccines was empirically obtained by serial passaging of wild-type yellow fever *Flavivirus*. However, such an approach is not acceptable nowadays, and the development of a rationally designed vaccine is necessary. Generating molecular infectious clones and mutating specific residues known to be involved in *Flavivirus* virulence constitute a powerful tool to promote viral attenuation. WNV membrane glycoprotein is thought to carry such essential determinants. Here, we identified two residues of this protein whose substitutions are key to the full and stable attenuation of WNV *in vivo*, most likely through inhibition of secretion and possible alteration of morphology. Applied to other flaviviruses, this approach should help in designing new vaccines against these viruses, which are an increasing threat to global human health.

## Abstract (precis)

West Nile virus (WNV) is a worldwide (re)emerging mosquito-transmitted *Flavivirus* causing fatal neurological diseases in humans. However, no human vaccine has been yet approved. One of the most effective live-attenuated vaccines was empirically obtained by serial passaging of wild-type yellow fever *Flavivirus*. However, such an approach is not acceptable nowadays, and the development of a rationally designed vaccine is necessary. Generating molecular infectious clones and mutating specific residues known to be involved in *Flavivirus* virulence constitute a powerful tool to promote viral attenuation. WNV membrane glycoprotein is thought to carry such essential determinants. Here, we identified two residues of this protein whose substitutions are key to the full and stable attenuation of WNV *in vivo*, most likely through inhibition of secretion and possible alteration of morphology. Applied to other flaviviruses, this approach should help in designing new vaccines against these viruses, which are an increasing threat to global human health.

## INTRODUCTION

West Nile virus (WNV) is a member of the *Flavivirus* genus. Most closely related to another genus member, Japanese encephalitis virus (JEV), but also similar to yellow fever virus (YFV), Zika virus (ZIKV), and dengue virus (DV), WNV is one of the most largely widespread neurotropic arthropod-borne viruses, causing severe neurological symptoms and even death (1, 2). Since its first isolation in Uganda in 1937, recurrent and unpredictable WNV outbreaks have been detected in humans throughout the world, generating health problems. Despite its global reemergence, however, there is currently neither treatment nor a human vaccine available to cure or prevent the disease (1). In the hope of aiding the development of innovative rationally designed vaccines, we focused our research on host-WNV molecular interactions and particularly viral particle assembly in the endoplasmic reticulum (ER) of the infected cell.

WNV possesses two structural glycoproteins, the membrane protein (M), processed from a precursor protein (prM), and the envelope protein (E) (3). While the E protein mediates interactions between host cellular factors and the virus for attachment and penetration, prM supports E during folding and shields it from causing premature fusion in the acidic secretory pathway (4). As with other flaviviruses, WNV assembly occurs in the endoplasmic reticulum (ER) and requires interactions between prM, E, and the nucleocapsid (5, 6). Following assembly, nascent virions bud into the ER lumen and are then translocated to the Golgi apparatus via trafficking vesicles (7). Once in the *trans*-Golgi network (TGN), prM is cleaved by the cellular furin, leading to the release of the pr peptide in the neutral-pH extracellular environment and the formation of mature and infectious M-containing virions (8). Although *Flavivirus* assembly mechanisms have been largely investigated (9, 10), it is still unclear how nascent virions engage the host cell secretory pathway in order to exit the ER and reach the TGN, to then be released at the cell surface.

**FIG 1:**
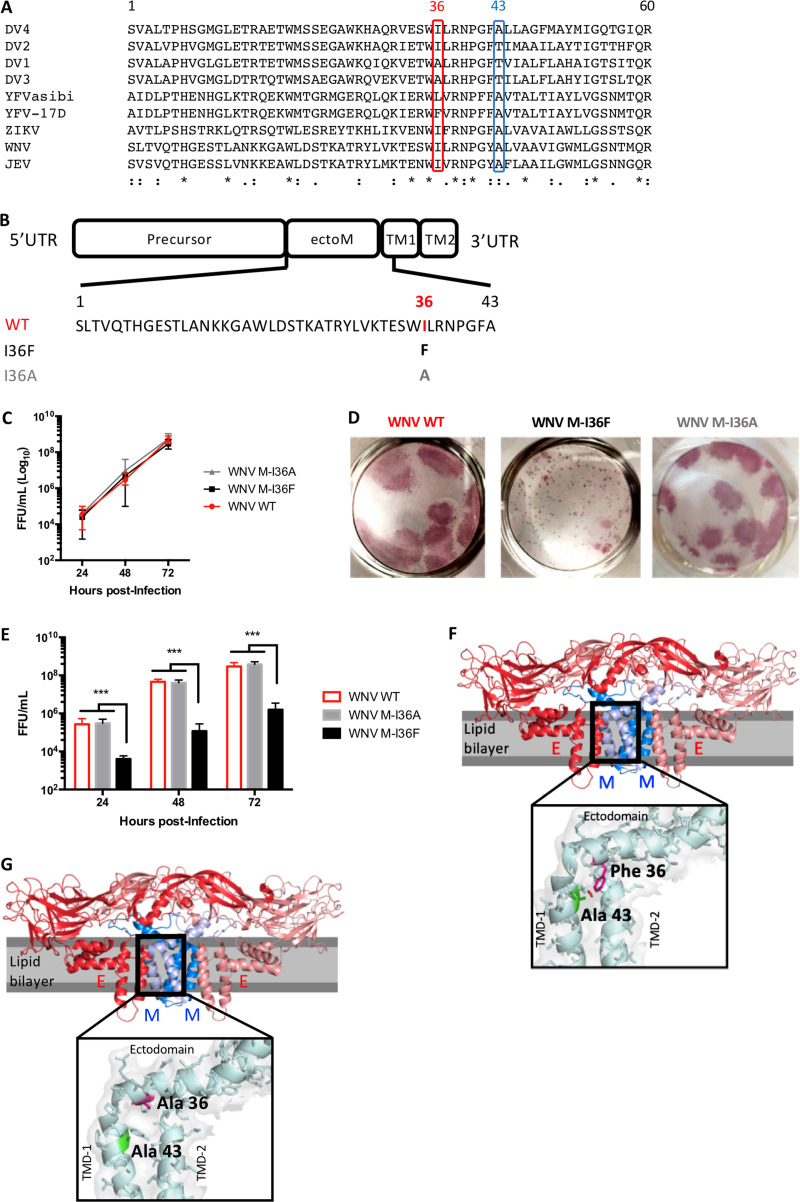
The nature of the M-36 residue impacts the WNV infectious cycle by potentially disrupting the M protein 3-dimensional structure. (A) Sequence comparison of the M protein ectodomain and TM1 from different flaviviruses. The location of residue 36 is indicated in red and that of residue 43 in blue. Accession numbers: DV4, MK506266.1; DV2, MK506264.1; DV1, MK506262.1; DV3, MK506265.1; YFV strain Asibi, AY640589; YFV strain 17D, MN708489.1; Zika virus, MG827392.1; WNV, AF481864.1; JEV, KF907505.1. (B) WNV membrane protein precursor (prM) organization, showing the sequences of the ectodomain (ectoM) and part of transmembrane domain 1 (TM1). The residue at position 36 is indicated in red for WT virus, black for M-I36F, or gray for M-I36A. (C) Viral stocks were collected from C6/36 cell supernatants at the times indicated and titrated by focus-forming assay (FFA) in Vero cells. No statistical difference was observed. (D) Focus morphology of wild-type WNV and M-I36F and M-I36A mutant viral stocks collected from C6/36 supernatants, observed on Vero cells. Vero cells were infected with the indicated virus, and foci were observed at 48 h postinfection. (E) Growth curves of wild-type, M-I36F, and M-I36A WNV. SK-N-SH cells were infected with the indicated virus at a multiplicity of infection (MOI) of 1, and cell supernatants were collected at indicated times for quantitation of virus titers by FFA using Vero cells. (F) Structure of M-E mature heterodimers (PDB accession number 5WSN). The inset zooms into the A43-F36 contact, with F36 highlighted in pink and A43 in green. The F36 aromatic ring (in red) clashes with the side chain of the A43 located in TMD-1. (G) Same as panel F with alanine at position M-36. The inset zooms into the A36-A43 contact, with A36 highlighted in pink and A43 in green. No clash between A36 and A43 was observed. The image was generated using PyMOL. The data are representative of 3 independent experiments, and error bars indicate standard deviation (SD). *, *P* < 0.05; **, *P* < 0.01; ***, *P* < 0.001.

The flavivirus prM/M protein was recently shown to carry virulence determinants (11, 12). In the ectodomain of the M protein, residue 36 (M-36) was suggested to be essential for proper *Flavivirus* viral morphogenesis, although the underlying mechanisms had not been evaluated (11, 13, 14). It has been shown, however, that among the 32 amino acid differences identified between the yellow fever virus vaccine strain 17D (YFV-17D) and the wild-type YFV Asibi strain, there is only one mutation in M at position 36, L36F (15). Noticeably, the same L36F substitution is found in another YFV vaccine strain, the French neurotropic virus (FNV) (16).YFV L36F was shown to be partially responsible for the inability of YFV-17D to infect and disseminate in mosquitoes (14). Interestingly, in other flaviviruses M-36 is always occupied by a hydrophobic residue (Fig. 1A), either isoleucine (WNV, JEV, DV2, and DV4), alanine (DV1 and DV3), or leucine (YFV Asibi), and any substitutions of this M-36 residue always lead to a decrease in the production of virus-like-particles (VLPs) in mammalian cells (11, 13, 17). Additionally, we recently demonstrated that in JEV, substitution of isoleucine at M-36 for phenylalanine abolished infectious virus production by altering late steps of the viral cycle (11).

As specific recognition signals between cellular and viral components are required for viral particle assembly and egress, we investigated the possible involvement of residue M-36 in WNV assembly and secretion from the ER to the Golgi apparatus. We substituted the isoleucine residue for a phenylalanine at position 36 in the M protein of WNV, generating an attenuated virus that displayed an impaired secretion but that was not stable. We then introduced a sterically compensatory substitution in the same protein at position 43, M-A43G, and obtained the stabilized double mutant M-I36F/A43G. This double mutant retained the specificities of the single mutant, eliciting a fully protective immune response against a lethal wild-type (WT) challenge in mice. We thus identified the M-36 residue as a molecular determinant of virulence that is crucial for efficient secretion of newly synthesized virions and the M-43 residue that accommodates and stabilizes a substitution in M-36. Our study strongly suggests that export of newly formed WNV particles from the ER may depend on their morphology and identifies the M protein as a new target for the rational design of attenuated WNV strains to prevent WNV disease.

## RESULTS

### Mutation of M-36 affects the WNV infectious cycle by potentially altering the M protein 3D structure.

Mirroring the M-L36F mutation in the YFV 17D vaccine, we replaced isoleucine 36 of the WNV M protein with a phenylalanine (M-I36F) (Fig. 1B). The resulting mutant virus was successfully produced in C6/36 cells electroporated with genomic RNA synthesized *in vitro* (see Materials and Methods) (Fig. 1C), and contrary to the case for wild-type WNV, the M-I36F mutant displayed a smaller-focus phenotype in Vero cells, which is a potential attenuation marker (Fig. 1D, M-I36F). Conversely, substitution of the parental isoleucine 36 with an alanine (Fig. 1B, M-I36A) did not affect focus size (Fig. 1D, M-I36A). More importantly, we observed that only the M-I36F mutation impaired the WNV infectious cycle in mammalian neuronal SK-N-SH cells (Fig. 1E), suggesting that the nature of residue 36 is essential for efficient viral particle production in these cells. While isoleucine, alanine, and phenylalanine possess similar chemical properties, only the phenylalanine has an aromatic ring. To examine how M-I36F might physically impact interactions with neighboring amino acids, we mapped either WNV M-I36F or WNV M-I36A into the recently published JEV M protein 4.3-Å cryo-electron microscopy structure (18), revealing that M-I36F (Fig. 1F), but not M-I36A (Fig. 1G), clashes with an alanine residue (A43) located in the first transmembrane segment of M (TM1). Thus, while interactions between the two structural proteins E and M are seemingly conserved, M-I36F potentially disrupts the M protein three-dimensional (3D) structure such that steric hindrance is introduced between the phenylalanine aromatic ring and the side chain methyl group of A43.

### Compensatory mutation partially rescues the M-I36F mutant to the WT phenotype.

**FIG 2:**
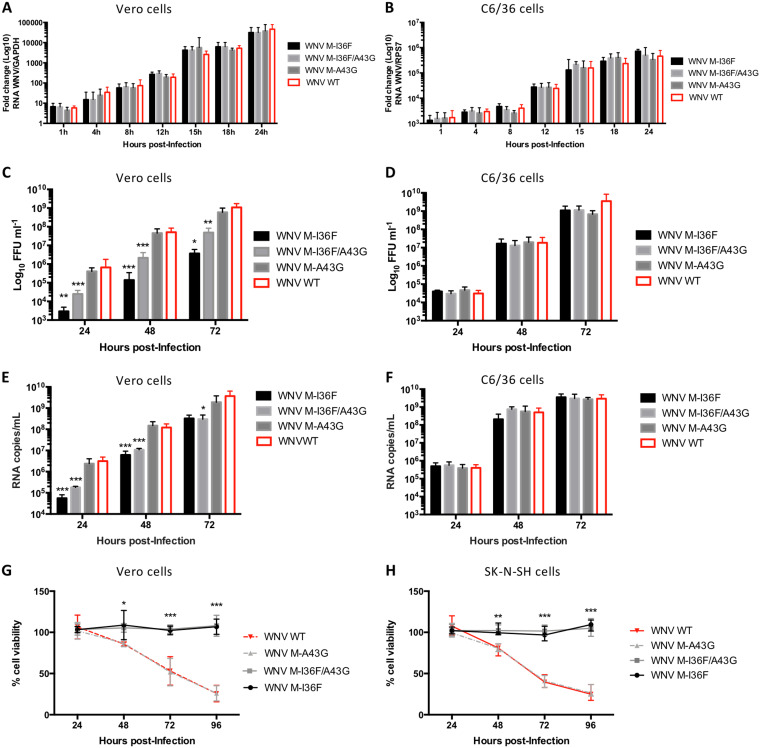
Phenotypical characterization of WNV M-I36F and M-A43G mutant viruses in vitro. (A and B) Viral stocks of WT WNV and mutants M-A43G, M-I36F, and M-I36F/A43G were used at an MOI of 1 to infect Vero cells (A) or C6/36 cells (B). At the indicated time points, cells were harvested and levels of WNV genomic RNA were quantified by RT-qPCR. (C to F) Growth curves and genome quantitation of wild-type, M-I36F, M-A43G, and M-I36F/A43G WNV produced in Vero cells. Vero cells (C and E) and C6/36 cells (D and F) were infected with the indicated viruses at an MOI of 1, and cell supernatants were collected at the indicated times for quantitation of virus titers by FFA using Vero cells (C and D) or for genome quantitation by RT-qPCR (E and F). (G and H) Cell viability. Vero (G) or SK-N-SH (H) cells were infected with the indicated viruses at an MOI of 1, cells were harvested at indicated times, and cell viability was evaluated using CellTiter Glo and represented as a percentage of noninfected control cells. The data are representative of 3 independent experiments, and error bars indicate standard deviation (SD). *, *P* < 0.05; **, *P* < 0.01; ***, *P* < 0.001.

To compensate for the potential clash between the aromatic ring of residue 36 and the side chain of residue 43, we substituted the original A43 by a residue that has no methyl group, namely, a glycine (M-A43G), in order to create more space, thereby generating the double mutant virus M-I36F/A43G. We recovered and amplified WNV M-I36F/A43G, M-A43G, and wild-type viruses from mosquito C6/36 cells electroporated with genomic RNA synthesized *in vitro* (see Materials and Methods). All viruses were found to form large foci on mammalian Vero cells (data not shown) and replicated similarly, as assayed for RNA production, in Vero (Fig. 2A) and C6/36 (Fig. 2B) cells, indicating that the M-I36F and M-A43G mutations alone or together did not affect genome decapsidation and replication in mammalian and mosquito cells. When comparing infectious particle production in Vero cell supernatants, however, the titers of M-I36F as well as M-I36F/A43G variants were largely lower than those of wild-type and M-A43G viruses (1.42 logs and 0.93 logs, respectively) (Fig. 2C), yet the M-I36F/A43G titers were significantly higher than that of M-I36F (Fig. 2C). Interestingly, when viruses were grown in C6/36 cells, no difference in titers was observed (Fig. 2D). Genetic stability of the mutant viruses was tested by 10 serial passages in Vero cells. Full-genome analysis of M-I36F/A43G passaged up to 10 times revealed the presence of both M-I36F and M-A43G and no other mutation along the genome, while M-I36F alone had already reverted to the WT sequence at passage 2 without compensatory mutation elsewhere in the genome (data not shown). A decrease in the amount of genomic viral RNA was observed over time in Vero cell supernatants infected with M-I36F or M-I36F/A43G mutants compared to wild-type or M-A43G viruses (Fig. 2E), which mirrored the decrease in infectious titers in mammalian cells (Fig. 2C) and corroborated a decrease in the number of secreted particles. No change in the amount of genomic viral RNA in mosquito cells infected either with wild-type or any mutant viruses was detected (Fig. 2F), again reflecting what we observed in terms of titers in these cells (Fig. 2D). Interestingly, neither M-I36F nor M-I36F/A43G mutant virus infection of mammalian cells induced any cell death (Fig. 2G [Vero cells] and H [SK-N-SH cells]), contrary to the case for the WT and A43G viruses. This result agrees with previous reports showing that residue M-36 can modulate the death-promoting activity of the M protein ectodomain of flaviviruses (19, 20). No cell death induction was observed for either the WT or any mutant viruses in infected C6/36 mosquito cells (data not shown). Altogether, these results indicated that the M-I36F mutation leads to an impaired WNV infectious cycle in mammalian cells, most likely due to the alteration of mutant viral assembly and/or egress, that can be partially rescued and completely stabilized by introduction of a second mutation relieving steric hindrance (M-A43G).

### The M-I36F mutation strongly inhibits efficient WNV secretion.

**FIG 3:**
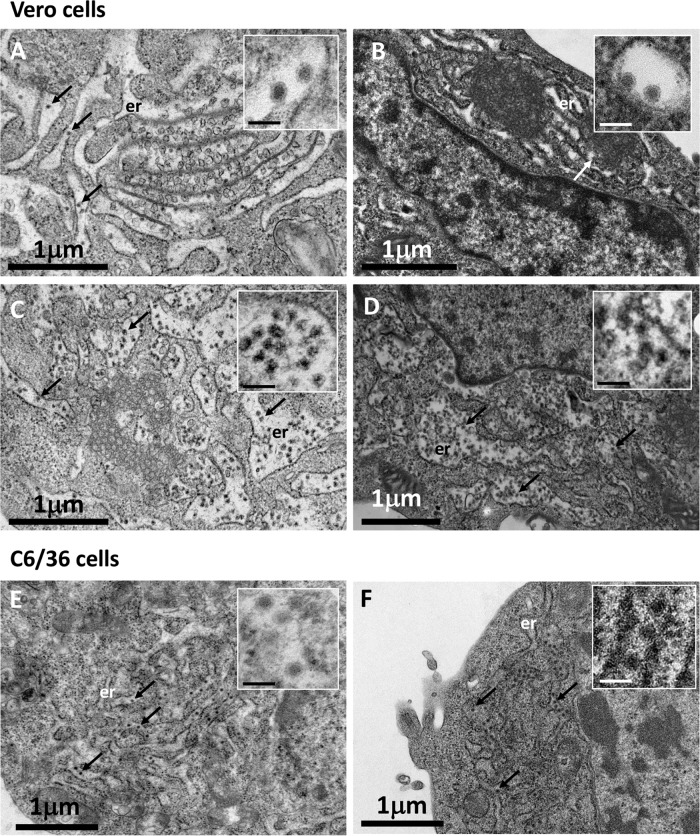
M-I36F and M-I36F/A43G mutant particles are retained within the ER lumen of infected mammalian cells but not in mosquito cells. (A to D) Vero cells were infected with wild-type WNV or WNV mutated at positions M-36 and/or M-43 at an MOI of 10 and examined by transmission electron microscopy at 24 h postinfection. (A) Vero cells infected with WT WNV. (B) Vero cells infected with mutated virus M-A43G. (C) Vero cells infected with mutated virus M-I36F. (D) Vero cells infected with double mutant virus M-I36F/A43G. (E and F) Mosquito C6/36 cells were infected with wild-type WNV or WNV mutated at positions M-36 and/or M-43 at an MOI of 10 and examined by transmission electron microscopy at 24 h postinfection. (E) C6/36 cells infected with WNV WT. (F) C6/36 cells infected with double mutant virus M-I36F/A43G. Examples of viral particles located in the ER lumen are indicated by arrows. Inset bars, 100 nm.

**FIG 4:**
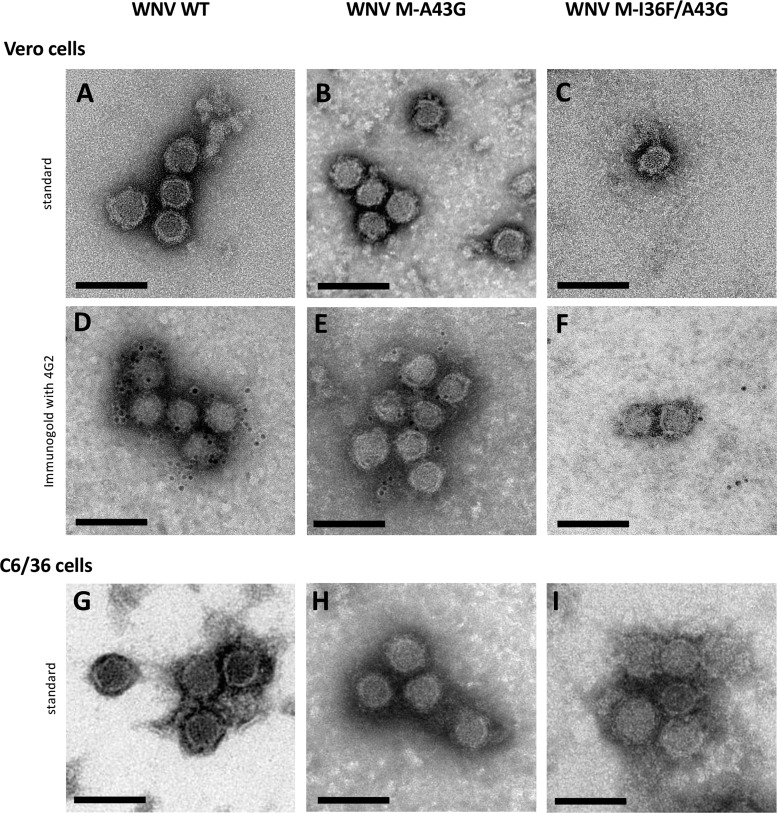
Secreted M-I36F/A43G mutant virions display an altered morphology only when produced in mammalian cells. (A to F) Wild-type and mutated viral particles collected from supernatants of Vero cells infected at an MOI of 10 for 24 h, were concentrated and purified. (A to C) Particles were negatively stained with uranyl and observed by transmission electron microscopy. (A) WT WNV particles. (B) WNV M-A43G particles. (C) WNV M-I36F/A43G particles. (D to F) Viral particles were labeled by immunogold with an anti-protein E pan-flavivirus antibody (MAb 4G2) and observed by transmission electron microscopy. (D) WT WNV particles. (E) WNV M-A43G particles. (F) WNV M-I36F/A43G particles. (G to I) Wild-type and mutated viral particles collected from supernatants of C6/36 cells infected at an MOI of 10 for 24 h were concentrated and purified. Particles were negatively stained with uranyl and observed by transmission electron microscopy. (G) WT WNV particles. (H) WNV M-A43G particles. (I) WNV M-I36F/A43G particles. Bars = 100 nm.

As only a few M-I36F/A43G and M-I36F mutant particles were found in the supernatant of mammalian cells, we wondered whether the M-I36F mutation could interfere with proper budding and/or secretion of the viral particles. We examined mammalian cells infected with the different viruses by electron microscopy (Fig. 3). Specific subcellular ultrastructural changes associated with the presence of each virus were observed in ultrathin sections of Vero cells infected with either wild-type or mutant viruses (Fig. 3A, B, C, and D). Relatively few wild-type and M-A43G viral particles were observed within the cells, with the occasional particle found in the ER, indicating that the virions are secreted normally (Fig. 3A and B, arrows). On the other hand, in the same cell type, infection with the M-I36F or M-I36F/A43G virus induced large ER swelling and massive accumulation of newly formed viral particles within the ER and ER-derived vesicles (Fig. 3C and D, arrows). No such impairment of particle secretion with either mutant was observed in infected mosquito cells (Fig. 3E and F). Importantly, the M-I36F and M-I36F/A43G mutant particles were released into the ER lumen of the infected mammalian cells and not retained at the ER membrane, indicating that assembly and budding steps still occurred in the presence of the M-I36F mutation alone or associated with M-A43G (Fig. 3A to D, zooms). The overall aspect of WNV M-I36F and M-I36F/A43G mutant particles seemed irregular compared to that of wild-type and M-A43G mutant viruses in mammalian cells (Fig. 3A to D, zooms), suggesting that WNV morphology was potentially altered by the M-I36F mutation. Indeed, the few M-I36F/A43G virions secreted into the supernatant of mammalian cells at 24 h postinfection directly observed by standard negative-staining electron microscopy seemed to display an altered morphology, although the nucleocapsid and the lipid envelope were still well delineated (Fig. 4C). While we were unable to obtain any image for the M-I36F mutant due to an insufficient number of secreted particles, those of wild-type and mutant M-A43G virions showed typical characteristics of flavivirus particles (Fig. 4A and B). The specificity of the particles produced from infected mammalian cells was confirmed using immunogold labeling with monoclonal antibody (Mab) 4G2, and the presence of WNV E protein at the surface of wild-type, M-A43G, or M-I36F/A43G virions was unambiguously observed (Fig. 4D to F), although less labeling was found at the surface of the double mutant virions. On the other hand, wild-type and mutant M-I36F, M-A43G, and M-I36F/A43G virions collected from supernatants of mosquito cells all displayed the morphological characteristics of classic flaviviruses (Fig. 4G to I). Taken together, these data suggest that the M-I36F mutation affects virion secretion, possibly by altering WNV morphology, only in mammalian cells.

### The atypical particle morphology of the M-I36F/A43G variant impacts the WNV antigenic profile.

**FIG 5:**
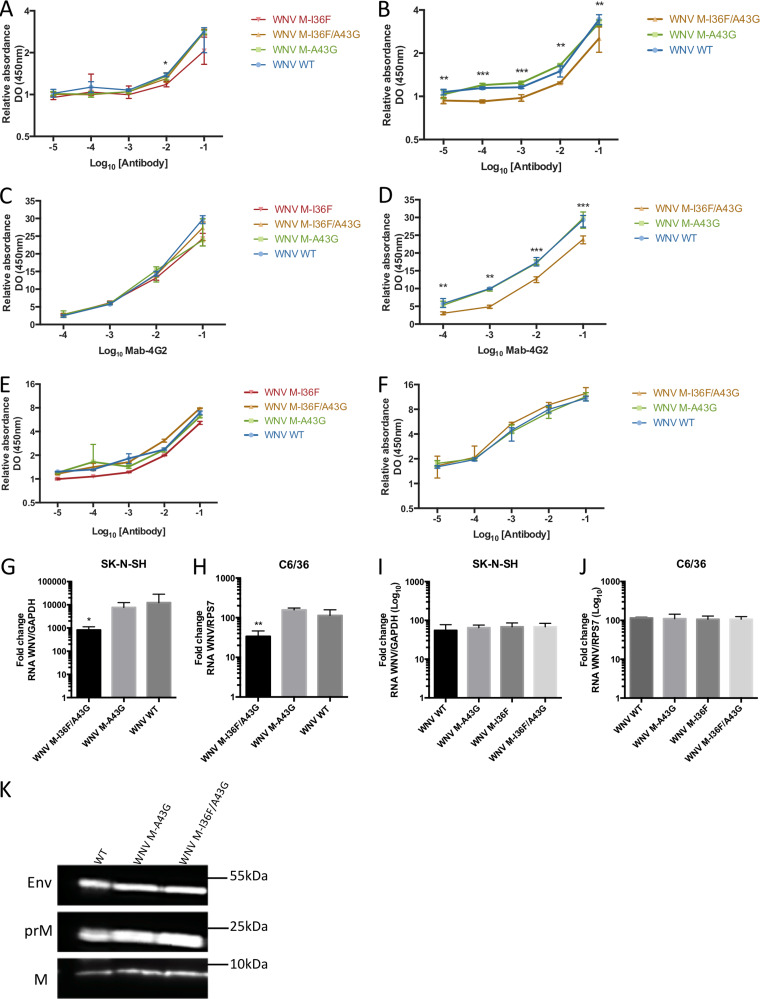
Effects of the M-I36F mutation on the WNV antigenic profile. (A and B) Wild-type and mutated WNV surface epitope exhibition was analyzed by direct ELISA. Two hundred nanograms of different UV-inactivated viruses collected from C6/36 cells (A) or Vero cells (B) was applied and tested with increasing concentrations of MAb 4G2. (C and D) Same as panels A and B, respectively, using indirect noncompetitive ELISA. (E and F) Same as panels A and B, respectively, but with increasing concentrations of polyclonal anti-WNV antibodies. (G and H) The infectious capacity of mutant virus M-I36F/A43G is impaired when the virus is produced in mammalian cells. SK-N-SH and C6/36 cells were placed at 4°C for 1 h and then infected at an MOI (amount of viral genomic RNA) of 10 for 1 h at 4°C with the indicated viruses produced in mammalian cells. (G) SK-N-SH cells were collected, and viral genomes attached to the cell-surface were quantified by RT-qPCR. (H) Same as panel G but with C6/36 cells. (I and J) The infectious capacity of WNV mutated at position M-I36F alone or associated with M-A43G is not affected when the virus is produced in mosquito cells. SK-N-SH and C6/36 cells were placed at 4°C for 1 h and then infected at an MOI (amount of viral genomic RNA) of 10 for 1 h at 4°C with the indicated viruses produced in mosquito cells. (I) SK-N-SH cells were collected, and viral genomes attached to the cell-surface were quantified by RT-qPCR. (J) Same as panel I but with C6/36 cells. (K) The levels of the E, immature prM, and mature M glycoproteins were tested under denaturing conditions by Western blotting using a polyclonal anti-WNV antibody. The same amount of viral RNA was loaded in each well. The histograms indicate the median value and the interquartile range determined from triplicates of three independent experiments. *, *P* < 0.05; **, *P* < 0.01; ***, *P* < 0.001.

Thus, a potential modification(s) of M protein structure caused by the M-I36F mutation might lead to altered viral particle morphology with irregularly shaped mutant virions. We reasoned that such atypical morphology of the mutant particles may impact the virion antibody recognition. We therefore first evaluated the recognition profiles of wild-type and mutant virions by direct enzyme-linked immunosorbent assay (ELISA) (Fig. 5). While viruses produced in C6/36 cell supernatants are all similarly recognized by MAb 4G2, which binds specifically the fusion loop of the E protein (21, 22) (Fig. 5A), recognition of M-I36F/A43G virus collected in the supernatant of Vero cells is significantly decreased, by approximately 1.2-fold, for any antibody dilution compared to that of the wild-type and the M-A43G mutant (Fig. 5B). A similar significant decrease (ranging from 1.2- to 2-fold, depending on the antibody dilution) in recognition of WNV M-I36F/A43G produced in Vero cells by MAb 4G2 was obtained using indirect noncompetitive ELISA (Fig. 5D), while viruses produced in C6/36 cell supernatants were all similarly recognized (Fig. 5C). Importantly, no difference in recognition by polyclonal anti-WNV antibody of wild-type and mutant viruses produced in either insect (Fig. 5E) or mammalian (Fig. 5F) cells was observed, indicating that despite a slightly significantly decreased recognition of the protein E fusion loop, the general antigenic properties of WNV M-I36F/A43G mutant virus are conserved.

WNV surface epitopes are essential for both efficient recognition and cell attachment, and the proper folding of the E protein chaperoned by the M protein in the prM-E complex plays a critical role in these processes. We therefore tested the infectious capacity of our mutant and wild-type viruses under conditions allowing viral binding, but not internalization, to SK-N-SH mammalian cells or C6/36 mosquito cells by evaluating viral genomic RNA associated with the cell surface (Fig. 5G and H, respectively). Comparing viruses produced in mammalian cells and assayed at the surface of SK-N-SH or C6/36 cells, levels of M-I36F/A43G RNA were reduced by around 1 log compared to those of the wild-type and M-A43G viruses (Fig. 5G and H), indicating that the WNV double mutant M-I36F/A43G has impaired binding to host cells. Conversely, wild-type, M-I36F, M-A43G, and M-I36F/A43G viruses produced in insect cells showed no difference in RNA levels (Fig. 5I and J). To confirm that the decreased infectious capacity of M-I36F/A43G mutant virus was not simply due to a lack of maturation, we tested for the presence of the immature (prM) and mature (M) forms of the membrane glycoprotein at the surface of wild-type or mutant virions collected from Vero cell supernatants by Western blotting (Fig. 5K). The presence of similar levels of prM and M for wild-type and mutant viruses alike suggested that all viruses undergo a similar maturation process. Taken together, the above results support the notion that virions harboring the M-I36F mutation have a possibly altered morphology, while the main WNV antigenic properties are conserved.

### *In vivo* effects of the WNV M-I36F and/or M-A43G mutation.

**FIG 6:**
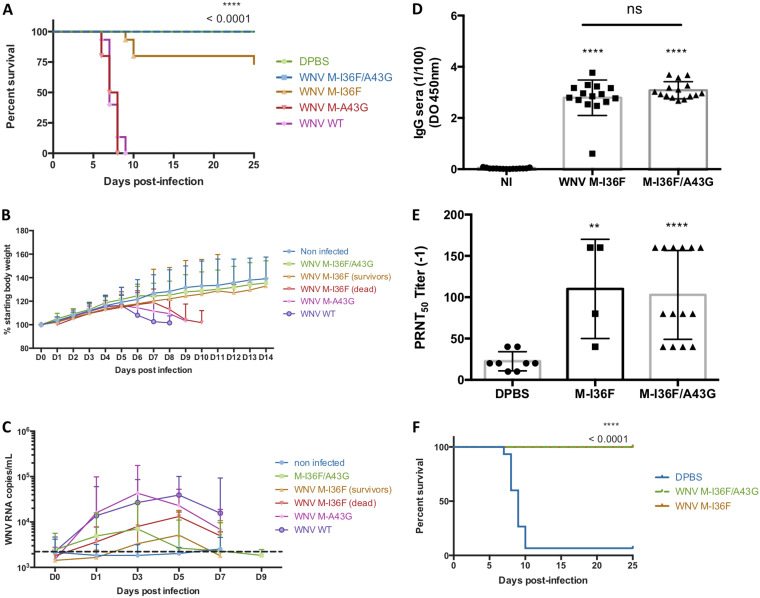
Combined M-I36F and M-A43G mutations highly attenuate WNV and elicit a WNV-specific humoral response in a mouse model. (A) Survival curves of 3-week-old BALB/c mice inoculated with 50 FFU of the indicated viruses by the intraperitoneal route. (B) Mouse growth curve. Mouse weight was measured every day postinfection and is represented as a percentage of the starting body weight. (C) Viral load in mouse blood. Viral RNA loads were quantified by RT-qPCR. The dashed line indicates the detection limit. (D and E) WNV-specific IgG and neutralizing antibodies were measured by ELISA and 50% plaque reduction/neutralization titer (PRNT_50_), respectively. (F) Survivor mice were challenged with 1,000 FFU of wild-type WNV at day 28 postinfection. Mice were monitored for clinical symptoms and mortality for 25 days. The data are representative of at least two independent experiments, and error bars indicate the SD *, *P* < 0.05; **, *P* < 0.01; ***, *P* < 0.001.

The *in vitro* properties of the WNV M-I36F and M-I36F/A43G mutants encouraged us to test their phenotypes *in vivo*. We first assessed pathogenicity in a well-established mouse model of WNV-induced encephalitis (23). In contrast to the high mortality rate observed among mice infected with either wild-type or M-A43G WNV (in which all 15 animals died), only 4 of 15 WNV M-I36F-infected mice died after being infected, while all mice infected with M-I36F/A43G survived (Fig. 6A). As expected, the wild-type-, M-A43G-, and M-I36F-infected mice that died presented rapid weight loss beginning at day 6 postinfection (Fig. 6B, purple, pink, and red curves). Conversely, rather than weight loss, we observed normal weight gain among mice that survived the infection (Fig. 6B, yellow and green curves). To investigate whether the WNV M-I36F and M-I36F/A43G mutants were attenuated due to a less effective viral dissemination, we collected blood samples every other day following the infection, for 10 days, and assayed for viral load. The results showed that viral loads peaked at day 3 for both the M-A43G and M-I36F/A43G mutants but peaked slightly later, at day 5 postinfection, for the wild-type and the M-I36F mutant (Fig. 6C). At day 3 or 5 postinfection, however, blood viral loads of M-I36F and M-I36F/A43G survivors were 3.4- or 14.7-fold and 4- or 7.6-fold lower, respectively, than that of wild-type-infected mice (Fig. 6C). Taken together, these data indicate that the M-I36F/A43G and M-I36F viruses are rapidly cleared following infection. Sequence analyses of the entire M-I36F/A43G mutant genome collected from blood samples revealed no reversion to wild type and no compensatory mutation (data not shown). This contrasts dramatically with the results of sequencing M-I36F viral genomes harvested from mice that did not survive the infection, which showed a reversion to the parental genotype (M-I36) (data not shown). Altogether, these results demonstrate that the M-I36F mutation strongly attenuates WNV *in vivo* and that the presence of the M-A43G mutation allows for stable retention of M-I36F attenuation without negative impact. Next, we investigated the immunogenic profile of the M-I36F and M-I36F/A43G mutants in mice (Fig. 6D and E). A single intraperitoneal injection of either M-I36F or M-I36F/A43G into adult BALB/c mice induced high levels of both WNV-specific IgG and neutralizing antibodies at day 27 postinfection (geometric mean titer = 102.86 and 110, respectively) (Fig. 6D and E, respectively). Induction of a remarkably robust neutralizing-antibody response to WNV M-I36F and M-I36F/A43G in mice led us to explore the protection afforded against a lethal challenge with the wild-type strain. Mice that had survived infection with M-I36F or M-I36F/A43G virus, or control mice injected with phosphate-buffered saline (PBS), were infected with 1,000 focus-forming units (FFU) of wild-type WNV. Not surprisingly, all but one control mouse developed symptoms upon intraperitoneal challenge and died from the infection around 8 days postinfection. Importantly, none of the mice that had been injected with a single dose of the M-I36F/A43G mutant virus exhibited symptoms after being challenged with wild-type WNV, and all survived the infection (Fig. 6F, green curve). Such protection was also conferred to mice that had survived M-I36F mutant infection when the mutant virus did not revert (Fig. 6F, yellow curve). These results demonstrate that the M-I36F and M-A43G mutation combination confers both full attenuation of WNV and full protection against wild-type WNV challenge in mice.

## DISCUSSION

WNV is one of the most widely distributed arboviruses in the world (24). Responsible for encephalitis in equids and humans, this virus recently reemerged in Europe during the 2016 and 2018 summers, causing an unusual number of human cases, including almost two hundred deaths (25). Currently no treatment or human vaccine is available. We focused our study on one of the two surface glycoproteins, namely, the M membrane protein. Previously the M protein has been described as being essential for viral maturation and fusion steps (8). In the present study, we now reveal a role for this protein in WNV secretion by demonstrating that substitution of a phenylalanine for an isoleucine at position 36 of the M protein (M-I36F) drastically decreases virion secretion out of the mammalian ER to the Golgi apparatus. This substitution appears to perturb the morphology of newly formed virions, which leads to massive ER accumulation of WNV progeny, while the same mutation does not seem to affect the morphology or secretion of virions during mosquito cell infection. We propose that the M-I36F substitution may cause steric hindrance that could directly affects the structure of the M protein. Importantly, in a mouse model of WNV infection, we found that the M-I36F mutant virus is strongly attenuated, resulting in the survival of most of the mice upon infection and in the production of neutralizing antibodies by all of the survivors. As this mutation is unstable, stochastically reverting to wild type within the mice, we demonstrate that we could achieve full stabilization of M-I36F by introducing a sterically compensatory substitution in the M protein, M-A43G. We found that this stable double mutant (M-I36F/A43G) retained the specific impaired secretion, viral attenuation, and resultant production of neutralizing antibodies found in the mice infected with the single M-I36F virus.

### A clash between the M-36 and M-43 residues likely affects viral secretion and morphology.

Our results indicate that the M-I36F mutation directly affects the WNV infectious cycle most likely by causing a clash between the phenylalanine residue and the alanine at position M-43 located in TM1. Similarly, substitution of residue M-36 also impairs JEV late steps, DV production, and even YFV dissemination (11, 13, 14), suggesting that the nature of the amino acid M-36 is crucial for *Flavivirus* particle production. The chemical properties of phenylalanine are close to those of the residues mainly found at position M-36 of other flaviviruses (A, I, or L). Therefore, its introduction may not change the nature of the M protein helical region. However, introduction of a larger residue at position M-36 could lead to a steric hindrance. Due to its volume, phenylalanine at position 36 might repel the M-A43 residue, which could disturb TM1 alpha helix positioning. The M protein TM1 domain is known to be involved in viral assembly and secretion of *Flavivirus*, likely through its interaction with the TM2 domain (26–28). Interestingly, disruption of JEV TM1/TM2 interactions has been shown to decrease the secretion of JEV virus-like-particles (26). Our observations indicate that introduction of a large residue such a phenylalanine at position M-36 is disruptive, while that of a small residue such as an alanine is not. By potentially repulsing residue M-43 in TM1, phenylalanine could disturb interactions critical to proper viral biogenesis. We found that a compensatory substitution of the M-A43 residue for a glycine in TM1, a residue that does not have a side chain, partially restores a wild-type phenotype with a significantly greater quantity of viral particles secreted in cell culture supernatants, seemingly by relieving the clash.

### The M-36 residue is crucial for correct virus secretion by potentially altering viral morphology.

Membrane curvature is known to be essential for *Flavivirus* morphology (29). Because our data potentially associate the M protein with viral morphology and due to its location at the surface of the particle and its interactions with the ER-derived lipid membrane, it is possible that the M protein ectodomain mutation could directly affect the membrane curvature, thus resulting in abnormal viral budding. It has been shown that *Flavivirus* heterodimers of prM and E assemble laterally and that their association induces ER membrane curvature in an isometric network (30). Therefore, introducing an aromatic phenylalanine residue that directly faces the ER membrane could, due to modified hydrophobic interactions, cause partial insertion of the M ectodomain into the ER membrane. As secretion and morphology of the mutant M-I36F and M-I36F/A43G virions from infected mosquito cells are normal, interactions between the M ectodomain and the ER membrane are probably unaffected by the I36F mutation in these cells. Since mosquito cells grow at a lower temperature than mammalian cells (28°C versus 37°C), one hypothesis could be that temperature by itself might be responsible for the M-I36F mutant phenotype by affecting viral assembly. However, preliminary data we obtained with the WNV M-I36F mutant from mammalian cells cultivated at 32°C show that secretion is still altered compared to that of wild-type virus (data not shown).

We demonstrated that it is the M-I36F mutation alone that causes alteration of the WNV infectious cycle, as the M-A43G mutation alone has no effect. Intriguingly, it has been shown that the substitution in DV1 virus-like particles of the M-L36 residue by an alanine increases prM and E glycoprotein interactions, leading to particle condensation (17). Such a condensation may particularly affect the “breathing” of the particles, a dynamic phenomenon dependent on temperature that ensures the metastability of E dimers and transient exposure of this protein’s buried domains. Since the WNV M-I36A substitution does not affect the viral cycle, we cannot conclude whether or not such mutation alters the dynamic phenomenon of WNV particle “breathing.”

Our results also indicate a lack of virus-dependent cell death in mammalian cells infected with M-I36F and M-I36F/A43G mutants, in contrast to the case for WT and M-A43G viruses. Residue M-36 is located in the proapoptotic domain (“apoptoM”) of the M protein that was shown to be essential for apoptosis induction by flaviviruses (19, 20). The M-I36F substitution in the M protein of YFV vaccine strain 17D abolished apoptosis induction, pointing to a crucial role for the M-36 residue. Importantly, the proapoptotic activity of the M protein ectodomain has been associated with its transport along the secretory pathway and its localization in a post-Golgi compartment (20). The massive accumulation of WNV M-I36F mutants within the ER and the ensuing inhibition of particle secretion that we observed probably hampered the export of the M ectodomain from the Golgi apparatus to the plasma membrane, abolishing apoptosis initiation. Noticeably, introduction of the M-A43G mutation in the M-I36F mutant, while partially rescuing the WNV life cycle, did not restore death-promoting activity.

### The M-I36F and M-A43G mutations together fully attenuate WNV *in vivo*.

We demonstrated that the altered virulence of our mutant virions drives a strong viral attenuation in a mouse model of WNV-induced encephalitis (23, 31, 32). Although the phenylalanine substitution at position M-36 was naturally selected for in the YFV vaccine strain 17D (15), reducing the ability of the virus to spread in the mosquito (14), the role of this mutation in the mammalian host has yet to be evaluated. The M-36 residue may represent a common virulence factor in *Flavivirus*, however, since it has been demonstrated that introduction of a M-I36F mutation in JEV leads to production of a live attenuated virus in a murine model (11). Although the quick reversion of M-I36F to wild type underlines the low stability of this mutation when it is alone, the simultaneous presence of the M-I36F and M-A43G mutations brings a stability to the resulting virus, without apparent reversion or compensatory mutation during the infection. Thus, the combination of these mutations leads to a double mutant virus with all the characteristics of a fully attenuated virus *in vivo*: suppression of lethality, limited weight loss, weak viremia, no neurological symptoms, and production of a neutralizing and protective immune response against a lethal challenge.

Curiously, the M-I36F mutation slightly alters antibody recognition by monoclonal antibody 4G2, while the general antigenic properties of the WNV M-I36F/A43G mutant virus are conserved (Fig. 5). MAb 4G2 has been characterized to bind specifically the fusion loop of the E protein (21, 22). As the fusion loop is buried in the E dimer at the particle surface, MAb 4G2 can bind this epitope only when the E dimer dissociates. The M-I36F mutation we introduced may impair the accessibility of the E fusion loop either by masking it or by blocking it in a “buried” state. However, MAb 4G2 can also bind to immature patches in partially mature particles. Interestingly, such partially mature particles are readily found in our cell culture supernatants, as evidenced by the presence of both prM and M proteins in all viruses (Fig. 5K). Importantly, the M-I36F mutation, while making virions less infectious, does not alter general antigenic recognition as shown both *in vitro* and *in vivo* (Fig. 5F and 6D and E). Protection against flaviviruses is correlated with a large production of neutralizing antibodies (33–35). Neutralizing antibodies are generally most efficient when directed against a specific amino acid sequence in domain III of the E glycoprotein (36). Our *in vitro* data indicate that M-I36F and M-I36F/A43G mutant viruses have kept highly immunogenic epitopes and that these mutations have not altered recognition of this E domain *in vivo*. On the other hand, antibodies directed against the domain II fusion loop of flaviviruses are generally poorly neutralizing and may lead to an increase in the antibody-dependent enhancement (ADE) phenomenon (37, 38). It would be interesting to evaluate whether the presence of M-I36F and M-A43G mutations, by decreasing recognition of the protein E fusion loop, may potentially reduce ADE.

Our study provides a robust proof of concept that M-I36F/A43G mutations may be used as a platform for the development of rationally designed attenuated WNV strains. Of course, a vaccine to prevent WNV infection must protect against all genotypes, especially in view of the recent emergence of lineage 2 neuroinvasive strains in Europe (39). It is known that prM and E proteins of one lineage cross protect against another lineage of WNV (40, 41). Therefore, it would be of interest to test the protective efficacy of our lineage 1 double mutant virus on a circulating strain of lineage 2. Live-attenuated vaccines against various virus infections have been empirically obtained by successive passages of wild-type virus strains and therefore may present significant risks of vaccine accidents. To cope with the spread of *Flavivirus* worldwide, the development of rational vaccine design approaches is critical. Our study opens new perspectives for the development of live-attenuated vaccines based on molecular alteration of virulence determinants in viral genomes produced from infectious clones.

## MATERIALS AND METHODS

### Cells.

Green monkey epithelial cells (Vero-E6) and human neuroblastoma-derived cells (SK-N-SH) were maintained at 37°C in Dulbecco’s modified Eagle medium (DMEM) (Life Technologies) supplemented with 10% heat-inactivated fetal bovine serum (FBS). Aedes albopictus C6/36 cells were maintained at 28°C in Leibovitz medium (L15; Life Technologies) supplemented with 10% FBS.

### Production of recombinant WNV.

The “two-plasmid” cDNA clone of WNV strain Israel 1998 produced in our lab (31) was used. Mutations M-I36F and/or M-A43G were directly introduced in pUC57-5′UTR-NS1 through PCR mutagenesis using primers 5′-AAAACAGAATCATGGTTCTTGAGGAACCCTGG-3′ and 5′-CCAGGGTTCCTCAAGAACCATGATTCTGTTTT-3′ (M-I36F) or primers 5′-ACCCTGGATATGGACTGGTGGCAGC-3′ and 5′-GCTGCCACCAGTCCATATCCAGGGT-3′ (M-A43G) (mutations are underlined).

A full-length infectious clone was produced as previously described (31), purified, and transcribed *in vitro* using the mMessage mMachine SP6 kit (Thermo Fisher Scientific). The resulting RNA was electroporated in C6/36 cells (400 V, 25μF, 800Ω) in Opti-MEM medium (Thermo Fisher Scientific). Cell culture supernatants were collected at 72 h postelectroporation and used to infect 10^7^ C6/36 cells. At 3 days postinfection, viral supernatants were amplified by infecting 5 × 10^7^ C6/36 cells for 3 days before collection and utilization as final viral stocks. Full-length viral genomes were sequenced from cDNA obtained by reverse transcription using the Superscript II reverse transcription kit (Invitrogen) according to the manufacturer’s instructions. cDNAs were then amplified by PCR using the Phusion high-fidelity kit (Thermo Fisher Scientific) and primers presented in Table 1.

**TABLE 1:** Primers used for the amplification and sequencing of the complete wild-type and mutant viral genomes

Forward oligonucleotides			Reverse oligonucleotides			Size (bp)
Name	Sequence	*T_m_* (°C)	Name	Sequence	*T_m_* (°C)	
11-32F	CTGTGTGAGCTGACAAACTTAG	55	780-797R	CAAGCCCCCTTCTTGTTC	58	787
501-520F	GACGGTAAATGCTACTGACG	56	1282-1300R	TTCCTTTGCCAAATAGTCC	55	800
1000-1018F	TTGGAAGGAGTGTCTGGAG	56	1781-1800R	AGTGTTGCTTGAAAATTCCA	56	801
1500-1520F	AAAGCTTGGAGAATATGGAGA	56	2278-2295R	ATGGACAGCCTTCCCAAC	58	796
2000-2017F	CATTGAACGACCTAACGC	55	2766-2783R	GTGAGGCGTTTAGGTGCT	56	784
2500-2519F	CAAGAGCTGAGATGTGGAAG	56	3281-3298R	AGTCAATCTCTACCCGGC	55	799
3001-3022F	GAATGTGACTCGAAGATCATTG	57	3777-3799R	CCACCATAAACACTGGTTGTATC	58	799
3501-3518F	CCTCGTGCAGTCACAAGT	56	4280-4297R	CAATGTCAAGCTCTGCCA	57	797
4000-4017F	CTAGCCCTGCTAACACCC	56	4781-4798R	CCTCTCCGCTCATCAAAG	57	799
4500-4519F	TTGGAAGATATGGATGCTCA	57	5277-5294R	ACAACCCTGGTTGGTGCT	59	795
5003-5020F	CCACTGGAACATCAGGCT	57	5783-5800R	TTGGGTACTCCGTCTCGT	57	798
5500-5520F	GCAAGAGGTTACATTTCCACA	58	6282-6299R	CACCTCCGGTCGTGGTAT	59	800
6001-6018F	AGAAATCCGTCGCAAGTT	56	6783-6800R	GAGAGCAGCAACATTCCG	58	800
6503-6520F	TGCCTGAGCACTTCATGG	59	7283-7300R	AACCGGGAACCATGTAGG	58	798
7031-7049F	CAACAGCCTGGTCACTGTA	55	7775-7792R	AGCGATCGACTTCGATGA	58	762
7500-7517F	ACGAGAAGCCGGAATTTT	57	8255-8275R	GGAGCAGCTCCATCTTCTCTA	59	776
8000-8017F	GTCCCGGACATGAAGAGC	59	8663-8681R	CATGGTTTTGAGAGGAGCC	58	682
8482-8499F	GGAAAACCCCTGCTCAAC	58	9283-9300R	CCAGCCAGCTGTGTCATC	59	819
9017-9034F	GGGGGGAATGTCACACTT	58	9782-9800R	TCATACCATCCAGTTGACG	55	784
9500-9517F	TTGTCACCTACGCCCTAA	55	10282-10300R	GGTTGATAGCCACCTGGAT	57	801
10002-10019F	AAGAGACCTGCGGCTCAT	58	10783-10800R	TTTCGCCCTGGTTAACAC	57	799
10504-10521F	AAAGTCAGGCCGGGAAGT	60	11011-11028R	ATCCTGTGTTCTCGCACC	57	525

### Antibodies.

Monoclonal antibody (MAb) 4G2 (anti-*Flavivirus* E protein) and horseradish peroxidase (HRP)-conjugated MAb 4G2 were purchased from RD Biotech (Besançon, France). Polyclonal anti-WNV was isolated from the intraperitoneal liquid of mice infected with WNV. The secondary antibody HRP-conjugated goat anti-mouse IgG was purchased from Bio-Rad Laboratories. Secondary gold-conjugated goat anti-mouse antibody was purchased from Aurion (Wageningen, Netherlands).

### M protein 3D structure.

M protein three-dimensional (3D) structure data were obtained from the PDB (PDB accession number 5WSN) and edited using the PyMOL program.

### qRT-PCR.

For quantitative reverse transcription-PCR (RT-qPCR), total RNA was extracted from samples using NucleoSpin RNA (Macherey-Nagel) according to the manufacturer’s instructions. The RNA standard used for quantitation of WNV copy number was produced as previously described (31). The quantitation of a given target RNA was performed using 2 μl of RNA and the Power SYBR Green RNA-to-CT 1-Step instrument (Thermo Fisher Scientific) according to the manufacturer’s instructions. The QuantStudio 6 Flex Real-Time PCR 384-well instrument (Thermo Fisher Scientific) was used to measure SYBR green fluorescence with the following program: reverse transcription at 48°C (30 min), followed by an initial PCR activation step at 95°C (10 min) and 40 cycles of denaturation at 95°C (15 s) and annealing at 60°C (3 0s). Results were analyzed using the CFX Manager software (Bio-Rad). Primers 5′-GCGGCAATATTCATGACAGCC-3′ and 5′-CGGGATCTCAGTCTGTAAGTC-3′ were used for viral genome quantitation. Target gene expression was normalized to the expression of glyceraldehyde-3-phosphate dehydrogenase (GAPDH) mRNA, which was measured using the primers 5′-GGTCGGAGTCAACGGATTTG-3′ and 5′-ACTCCACGACGTACTCAGCG-3′ (42).

### Titration.

Vero-E6 cells were seeded at 8 × 10^4^ cells per well in 24-well plates and incubated at 37°C for 24 h. Tenfold dilutions of virus in DMEM were added to the cells and incubated for 1 h at 37°C. Unadsorbed virus was removed, and then 1 ml of DMEM supplemented with 1.6% carboxymethyl cellulose (CMC), 10 mM HEPES buffer, 72 mM sodium bicarbonate, and 2% FBS was added to each well, followed by incubation at 37°C for 2 days. The CMC overlay was removed, and the cells were washed with PBS and fixed with 4% paraformaldehyde for 15 min, followed by permeabilization with 0.2% Triton X-100 for 5 min. Cells were then washed with PBS and incubated for 1 h at room temperature with anti-E antibody (4G2), followed by incubation with HRP-conjugated anti-mouse IgG antibody. The foci were revealed using the Vector VIP peroxidase substrate kit (Vector Laboratories) according to the manufacturer’s instructions.

### Analysis of the secreted particles by negative-staining electron microscopy and immunogold labeling.

The clarified viral supernatant was purified by polyethylene glycol precipitation followed by ultracentrifugation at 50,000 × *g* and 4°C for 2 h (Optima L-100 XP ultracentrifuge; Beckman) on an iodixanol gradient (OptiPrep; Sigma-Aldrich). Fractions of interest were then fixed with 2% (vol/vol) paraformaldehyde (Sigma, St. Louis, MO)–0.1 M phosphate buffer (pH 7.2) overnight. Formvar/carbon-coated nickel grids were deposited on a drop of fixed sample, left for 5 min, and rinsed three times with phosphate-buffered saline (PBS). After a single wash with distilled water, the negative staining was performed with three consecutive contrasting steps using 2% uranyl acetate (Agar Scientific, Stansted, UK), before analysis under a transmission electron microscope (1011; JEOL, Tokyo, Japan).

For immunogold labeling, grids coated with the sample were washed and further incubated for 45 min on a drop of PBS containing mouse monoclonal antibody against *Flavivirus* E protein (4G2; 1:10). After 6 washes with PBS, grids were incubated for 45 min on a drop of PBS containing 10-nm-gold-conjugated goat anti-mouse IgG (1:30; Aurion, Wageningen, Netherlands). Grids were then washed with 6 drops of PBS, postfixed in 1% glutaraldehyde, and rinsed with 2 drops of distilled water before being negatively stained and observed under the microscope as described above.

### Ultrastructural analysis of the infected cells by transmission electron microscopy.

24h-infected Vero or C6/36 cells were trypsinized, rinsed once in PBS, and gently resuspended in cold fixation buffer containing paraformaldehyde 4% (Sigma, St-Louis, MO), 1% glutaraldehyde (Sigma), 0.1 M phosphate buffer pH 7.3, for 24h. Cells were then placed in a mixture of (1:1) propylene oxide/Epon resin (Sigma) and left overnight in pure resin for samples impregnation. Cells were then embedded in Epon resin (Sigma), and blocks were allowed to polymerize for 48 h at 60°C. Ultrathin sections of blocks were obtained with a Leica EM UC7 ultramicrotome (Wetzlar, Germany). Sections were deposited on Formvar/carbon-coated nickel grids and stained with 5% uranyl acetate (Agar Scientific), 5% lead citrate (Sigma), and observations were made with a JEOL 1011 transmission electron microscope.

### Mouse experiments.

Three-weeks-old female BALB/c mice (Janvier) were housed under pathogen-free conditions in a level 3 animal facility. Protocols were approved by the Ethics Committee for Control of Experiments in Animals (CETEA) at the Institut Pasteur and declared to the French Ministry under no. 00762.02. Mice were infected intraperitoneally with 50 FFU of either wild-type, M-I36F, M-A43G, or M-I36F/A43G virus in 50 μl of Dulbecco’s PBS (DPBS) supplemented with 0.2% bovine serum albumin. Mice were followed daily postinjection; survival rate, weight loss, and clinical symptoms were monitored. Every 2 days postinfection (days 1, 3, 5, 7, and 9) blood samples obtained by puncture at the caudal vein were collected and tested for the presence of viral RNA. Mice that survived the infection were challenged with 1,000 FFU of wild-type virus diluted in 50 μl of DPBS plus 0.2% BSA at day 28 postinfection. Mouse mortality was followed over time. Blood was obtained by puncture at the caudal vein at day 27 postinfection and collected in tubes containing EDTA, and serum was separated after centrifugation at 4,000 × *g* for 10 min in order to perform ELISA and seroneutralization assays.

### Direct ELISA.

Viruses were purified by polyethylene glycol precipitation followed by ultracentrifugation at 50,000 × *g* and 4°C for 2 h (Optima L-100 XP ultracentrifuge; Beckman) on an iodixanol gradient (OptiPrep; Sigma-Aldrich). Fractions of interest were then UV inactivated. High-binding 96-well plates (Nunc) were coated with 2 μg/ml of purified and inactivated viruses in 100 μl of PBS–3% milk and 0.5% Tween 20 (PBS-milk-Tween) and incubated overnight at 4°C. Plates were washed five times with PBS containing 0.05% Tween 20. MAb 4G2, polyclonal anti-WNV antibodies, or sera obtained from mouse blood were serially diluted 10-fold (morphology analyses) or 2-fold (mouse experiments) starting at a 1:100 dilution in PBS-milk-Tween, added to plates, and incubated for 1 h at 41°C. After washing, plates were incubated with 100 μl of HRP-conjugated goat anti-mouse IgG diluted 1:10,000 in PBS-milk-Tween for 1 h at 41°C. Plates were washed again, and 200 μl of SigmaFast OPD (Sigma) substrate was added per well and left for 30 min following the manufacturer’s instructions. Luminescence was read on an EnVision 2100 Multilabel Reader plate reader (PerkinElmer, Santa Clara, CA, USA) at a wavelength of 450 nm.

### Indirect ELISA.

High-binding 96-well plates (Nunc) were coated with 5 μg/ml of polyclonal anti-WNV antibody in 100 μl of PBS-milk-Tween and incubated overnight at 4°C. Plates were washed five times with PBS containing 0.05% Tween 20, and 2 μg/ml of purified and inactivated viruses was added to plates and incubated for 2 h at 41°C. After washing, 100 μl of HRP-conjugated MAb 4G2 serially diluted 10-fold in PBS-milk-Tween was added to plates and incubated 1h at 41°C. Plates were washed, and 200 μl of SigmaFast OPD HRP substrate (Sigma) was added per well and left for 30 min following the manufacturer’s instructions. Luminescence was read on an EnVision 2100 Multilabel Reader plate reader (PerkinElmer, Santa Clara, CA, USA) at a wavelength of 450 nm.

### Seroneutralization assay.

Serum samples were 2-fold serially diluted in DMEM, with a starting dilution of 1:20. Each dilution was incubated with 50 FFU of wild-type WNV for 1 h under agitation at 37°C. The remaining viral infectivity was evaluated by focus-forming assay (FFA) on Vero-E6 cells. Sera collected from DPBS-injected mice served as negative controls. Neutralization curves were obtained and analyzed using GraphPad Prism 6 software. Nonlinear regression fitting with sigmoidal dose response was used to determine the dilution of serum that reduced the quantity of FFU by 50%.

### Statistical analysis.

Statistical analyses were performed using GraphPad Prism software. The nonparametric Mann-Whitney test was used to compared quantitative data, and log rank (Mantel-Cox) analysis was used for survival data analysis.
